# Carbazochrome sodium sulfonate is not effective for prevention of post-gastric endoscopic submucosal dissection bleeding: A retrospective study

**DOI:** 10.1007/s00464-022-09171-4

**Published:** 2022-03-07

**Authors:** Keitaro Takahashi, Takahiro Sasaki, Nobuhiro Ueno, Kyoko Uehara, Yu Kobayashi, Yuya Sugiyama, Yuki Murakami, Takehito Kunogi, Katsuyoshi Ando, Shin Kashima, Kentaro Moriichi, Hiroki Tanabe, Toshikatsu Okumura, Mikihiro Fujiya

**Affiliations:** Gastroenterology and Endoscopy, Division of Metabolism and Biosystemic Science, Gastroenterology, and Hematology/Oncology, Department of Medicine, Asahikawa Medical University, 2-1 Midorigaoka-higashi, Asahikawa, Hokkaido 078-8510 Japan

**Keywords:** Carbazochrome, Post-operative hemorrhage, Gastric neoplasm, ESD, Prevention

## Abstract

**Background:**

Carbazochrome sodium sulfonate (CSS) is conventionally administered to prevent post-endoscopic submucosal dissection (ESD) bleeding in many institutions, but research on its preventive efficacy is lacking. Therefore, we investigated the risk of post-ESD bleeding and the preventive efficacy of CSS administration.

**Methods:**

We retrospectively reviewed 304 lesions in 259 patients with gastric neoplasms who underwent ESD at Asahikawa Medical University Hospital from 2014 to 2021. In the CSS group, CSS 100 mg/day was intravenously infused with maintenance fluid replacement on postoperative days 0–2. The risk factors of post-ESD bleeding, including CSS administration, were investigated.

**Results:**

The overall rate of post-ESD bleeding was 4.6% (14/304). The univariate analysis showed that atrial fibrillation (Af), warfarin intake, heparin replacement, and tumor location in the lower third were significant risk factors for increasing the likelihood of postoperative bleeding. In the multivariate analysis, Af (odds ratio [OR] 3.83, 95% CI 1.02–14.30; *p* < 0.05), heparin replacement (OR 4.60, 95% CI 1.02–20.70; *p* < 0.05), and tumor location in the lower third of the stomach (OR 6.67, 95% CI 1.43–31.00; *p* < 0.05) were independent factors for post-ESD bleeding. Post-ESD bleeding was observed in 5.2% (9/174) of the CSS group and 3.8% (5/130) of the non-CSS group, with no significant difference between the two groups (*p* = 0.783). Additionally, CSS was not shown to have preventive effects in groups with higher-risk factors, such as Af diagnosis, warfarin use, heparin replacement, and tumor location in the lower third of the stomach.

**Conclusion:**

CSS administration was not effective for the prevention of the post-ESD bleeding in the overall patient population as well as in higher-risk patients. This suggests that the administration of CSS for post-ESD bleeding prevention may need to be reconsidered.

Endoscopic submucosal dissection (ESD) has been widely used for treating gastric neoplasms. ESD provides a high *en bloc* resection rate and overall good prognosis, although one of its major complications is postoperative bleeding [1]. The Japan Gastroenterological Endoscopy Society recommends use of hemostatic forceps to coagulate remnant vessels as well as the administration of a proton pump inhibitor (PPI) or an H2-histamine receptor antagonist to prevent post-ESD bleeding [2]. However, postoperative bleeding continues to occur in about 4–8% of gastric ESDs [3]. Various preventive methods, such as the polyglycolic acid shielding method, ulcer base closure, and use of polysaccharide hemostatic powder, have been implemented but have not significantly prevented post-ESD bleeding [4–6].

Carbazochrome sodium sulfonate (CSS) is a hemostatic agent that reduces capillary permeability and increases capillary resistance, resulting in shortened bleeding time [7, 8]. CSS has been used to treat bleeding of the gastrointestinal and respiratory tracts. The hemostatic effect of CSS has been shown in instances of hereditary hemorrhagic telangiectasia and total knee arthroplasty; however, CSS has not demonstrated hemostatic effects in instances of colonic diverticular bleeding [7–9]. Therefore, research regarding the hemostatic effects of CSS is currently controversial, especially in cases of gastrointestinal bleeding.

In the perioperative period of gastric ESD, CSS is conventionally administered to prevent post-ESD bleeding in many institutions, but there have been no reports verifying its preventive efficacy. Herein, we investigated post-ESD bleeding and the preventive efficacy of CSS administration.

## Materials and methods

### Study patients

We retrospectively reviewed a total of 328 consecutive lesions in 283 patients with gastric neoplasms who underwent ESD at Asahikawa Medical University Hospital from November 2014 to April 2021. We excluded five cases with non-neoplastic lesions, five cases with a gastric remnant, and fourteen cases with ESD discontinuation. Finally, 259 patients with a total of 304 gastric neoplasms were enrolled in the study. This study was approved by the institutional ethics committee of Asahikawa Medical University.

### Pre-ESD management of antithrombotic agents

In patients undergoing antithrombotic treatment, antithrombotic agents were withdrawn based on our hospital regulations and guidelines of the Japan Gastroenterological Endoscopy Society for patients undergoing antithrombotic treatment [10, 11]. Use of aspirin was discontinued for 3–5 days prior to the ESD procedure. Use of thienopyridine derivatives was halted 5–7 days in advance of the procedure. Warfarin was withdrawn for 3–4 days prior to the ESD procedure, and heparin replacement was withheld until the morning of the procedure. Direct oral anticoagulants (DOAC) and other antiplatelet agents were withdrawn on the morning of the procedure.

### ESD procedures

ESD was carried out by endoscopists at Asahikawa Medical University. A single-channel upper gastrointestinal endoscope (GIF-Q260J; Olympus Medical Systems, Tokyo, Japan) was used with a high-frequency generator (VIO-300D; Erbe Elektromedizin GmbH, Tübingen, Germany). The endoscopists selected an electrosurgical knife from FlushKnife BT-S (DK2620J; Fujifilm, Tokyo, Japan), a DualKnifeJ (KD-655L; Olympus Medical Systems, Tokyo, Japan), or an IT knife 2 (KD-610L; Olympus Medical Systems, Tokyo, Japan). Circumferential markings were made outside the tumor margin under the magnifying endoscopy with narrow-band imaging. Hyaluronic acid solution (Mucoup; Boston Scientific, Tokyo, Japan or Ksmart; Olympus Medical Systems, Tokyo, Japan) was injected into the submucosal layer to lift the surrounding mucosa. The mucosal incision was completed around the markings. Submucosal dissection was then initiated from the proximal side to the distal side and *en bloc* resection was performed. Immediately after ESD, a coagrasper (FD-412LR; Olympus Medical Systems, Tokyo, Japan) was utilized to discontinue hemorrhage from exposed blood vessels on the artificial ulcer. Ulcer base closure was not performed in any cases.

### Post-ESD clinical pathway

**Fig. 1:**
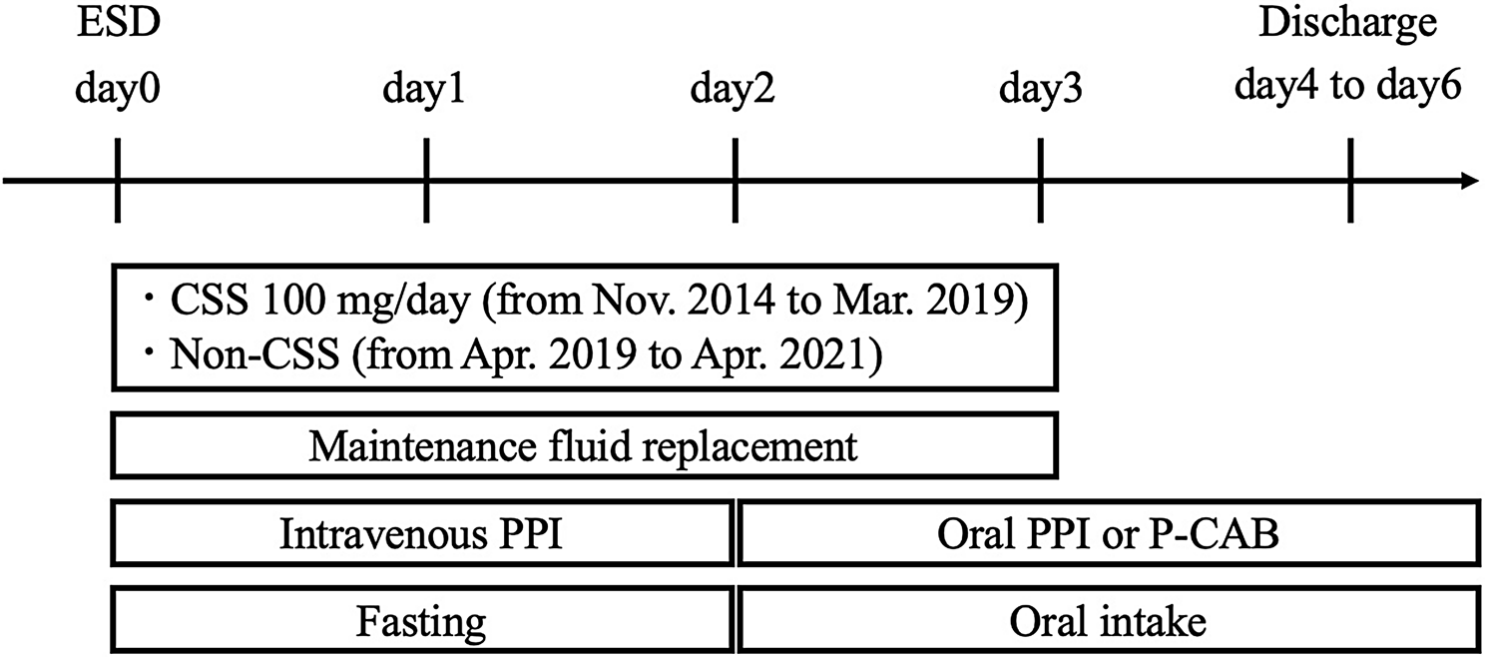
Post-endoscopic submucosal dissection clinical pathway. After endoscopic submucosal dissection (ESD), the patients were under fasting conditions, and maintenance fluid replacements were administered. Carbazochrome sodium sulfonate (CSS) (100 mg/day) was intravenously infused with maintenance fluid replacement on postoperative days 0–2 from November 2014 to March 2019. Maintenance fluid replacement without CSS was utilized from April 2019 to April 2021. Regarding the gastric acid-suppressing agents, intravenous proton pump inhibitor (PPI) was injected on postoperative days 0–1 and then the PPI or potassium-competitive acid blocker (P-CAB) was administered on and after postoperative day 2. Oral intake was reintroduced on postoperative day 2. The patients were discharged from our hospital on postoperative days 4–6.

A clinical pathway for our institution is shown in Fig. 1. After ESD, the patients maintained fasting conditions, and maintenance fluid replacement was administered. In our clinical pathway from November 2014 to March 2019, CSS was intravenously infused at 100 mg/day with maintenance fluid replacement on postoperative days 0–2. In April 2019, the clinical pathway was reviewed, and the infusion of CSS was stopped. Maintenance fluid replacement was utilized without CSS from April 2019 to April 2021. Regarding the gastric acid-suppressing agents, omeprazole sodium 40 mg/day (omeprazole; Nichi-Iko Pharmaceutical Company, Toyama, Japan) was injected on postoperative days 0–1, and either esomeprazole magnesium 20 mg/day (Nexium; Daiichi Sankyo Company, Tokyo, Japan) or vonoprazan fumarate 20 mg/day (Takecab; Takeda Pharmaceutical Company, Tokyo, Japan) was administered on and after postoperative day 2. The scheduled follow-up endoscopy was not performed in our clinical pathway. Instead, blood tests and physical examinations were completed on postoperative day 1, and an emergency endoscopy was performed on patients with hematemesis/melena or in patients with a decline in hemoglobin levels by ≥ 2 mg/dL. In cases where nothing abnormal was detected from the blood tests or physical examinations, antithrombotic agents were resumed on the day after the ESD procedure, and oral intake was reintroduced on postoperative day 2. The patients were discharged from our hospital on postoperative days 4–6.

### Post-ESD management for bleeding

Post-ESD bleeding is defined as hematemesis/melena required for endoscopic hemostasis or a decline in hemoglobin levels by ≥ 2 mg/dL. When post-ESD bleeding occurred, we performed emergency endoscopic hemostasis. After endoscopic hemostasis, the timing of antithrombotic agent resumption was determined by the endoscopists.

### Statistical analyses

All statistical analyses were performed using the R Project for Statistical Computing version 4.0.5 software program. Continuous variables were compared using Student’s *t* test, and nominal scale data were compared using Fisher’s exact probability test. Odds ratios (ORs) and 95% confidence intervals (95% CIs) were calculated to evaluate the strength of the influence of each individual variable. Selected variables with *p*-values < 0.05 in univariate analysis were included in the multivariate analysis. *p*-values of < 0.05 were considered to indicate statistical significance.

## Results

### Risk factors and rate of post-ESD bleeding

The overall rate of post-ESD bleeding was 4.6% (14/304). Regarding the risk of post-ESD bleeding, Table 1 shows the clinicopathological features of the post-ESD bleeding and non-bleeding groups. In the post-ESD bleeding group, characteristics including Af, warfarin intake, heparin replacement, and tumor location at the lower third of the stomach were significantly higher compared to the non-bleeding group (*p* < 0.05). Other factors (age, gender, antithrombotic agent use, potassium-competitive acid blocker (P-CAB) use, macroscopic type, histology, ulceration, depth of invasion, size of the resected specimen, tumor size, resection time, *en bloc* resection, R0 resection, and intraoperative perforation) did not differ between the post-ESD bleeding and non-bleeding groups.

**Table 1:** Clinicopathological features of the post-ESD bleeding and non-bleeding groups

	Post-ESD bleeding, *n* = 14	Non-bleeding, *n* = 290	*p* value
Age (years, mean ± SD)	71.1 (4.3)	73.4 (8.3)	0.309
Gender, *n* (%)			1
Male	10 (71.4)	172 (70.2)	
Female	4 (28.6)	73 (29.8)	
Co-morbidity			
Atrial fibrillation	4 (28.6)	20 (6.9)	0.018
Ischemic heart disease	0 (0)	12 (4.1)	1
Cerebrovascular disease	0 (0)	22 (7.6)	0.61
Chronic kidney disease on HD	0 (0)	4 (1.4)	1
Antithrombotic therapy, *n* (%)			
Aspirin	0 (0)	24 (8.3)	0.612
Thienopyridine derivatives	0 (0)	20 (6.9)	0.610
Cilostazol	0 (0)	10 (3.4)	1
Warfarin	3 (21.4)	5 (1.7)	0.004
DOAC	2 (14.3)	24 (8.3)	0.340
Others	1 (7.1)	23 (7.9)	1
Antithrombotic agent use, *n* (%)	6 (42.9)	86 (29.7)	0.371
Single	6 (42.9)	66 (22.8)	0.106
Doublet	0 (0)	15 (5.2)	1
Triplet	0 (0)	3 (1)	1
Heparin replacement	3 (21.4)	12 (4.1)	0.026
P-CAB	2 (14.3)	36 (12.4)	0.690
Tumor location, *n* (%)			
Lower third	12 (85.7)	132 (45.5)	0.004
Middle third	2 (14.3)	101 (34.8)	0.151
Upper third	0 (0)	56 (19.3)	0.081
Macroscopic type, *n* (%)			
Elevated	6 (42.9)	131 (45.2)	1
Flat/depressed	8 (57.1)	130 (44.8)	0.418
mixed	0 (0)	29 (10)	0.376
Histology			
Adenoma	4 (28.6)	54 (18.6)	0.316
Differentiated type	10 (71.4)	223 (76.9)	0.746
Undifferentiated type	0 (0)	13 (4.5)	1
UL +, *n* (%)	1 (7.1)	22 (7.6)	1
Depth			
SM2, *n* (%)	0 (0)	16 (5.5)	1
Resection size, mm (mean ± SD)	39.3 (16.9)	36.9 (12.5)	0.494
Resection size > 30 mm, *n* (%)	9 (64.3)	188 (64.8)	1
Tumor size, mm (mean ± SD)	19.1 (10.7)	16.5 (11.2)	0.396
Resection time, minutes (mean ± SD)	106.6 (66.4)	103.4 (71.6)	0.866
Resection time > 100 min, *n* (%)	8 (57.1)	117 (40.3)	0.268
*En bloc* resection, *n* (%)	14 (100)	281 (96.9)	1
R0 resection, *n* (%)	14 (100)	272 (93.8)	1
Intraoperative perforation, *n* (%)	0 (0)	5 (1.7)	1

*HD* hemodialysis, *DOAC* direct oral anticoagulants, *P-CAB* potassium-competitive acid blocker, *UL* ulceration, *SD* standard deviation

The results of the univariate and multivariate analyses for factors of post-ESD bleeding are summarized in Table 2. The univariate analysis showed significant risk factors and characteristics as follows: Af (OR 5.35, 95% confidence interval (95% CI) 1.12–20.75; *p* = 0.018), warfarin intake (OR 15.13, 95% CI 2.09–90.18; *p* = 0.004), heparin replacement (OR 6.24, 95% CI 0.99–28.35; *p* = 0.026), and tumor location at the lower third of the stomach (OR 7.14, 95% CI 1.55–66.93; *p* = 0.004). In the multivariate analysis, warfarin administration and heparin replacement showed high values of variance inflation factors. Therefore, warfarin administration was excluded from the analysis. As a result, Af (OR 3.83, 95% CI 1.02–14.30; *p* = 0.046), heparin replacement (OR 4.60, 95% CI 1.02–20.70; *p* = 0.046), and location of the tumor in the lower third of the stomach (OR 6.67, 95% CI 1.43–31.00; *p* = 0.016) were independent factors for post-ESD bleeding.

**Table 2:** Univariate and multivariate analyses for factors of post-ESD bleeding

	Univariate analysis			Multivariate analysis		
	OR	95%CI	*p* value	OR	95%CI	*p* value
Atrial fibrillation	5.35	1.12–20.75	0.018	3.83	1.02–14.30	0.046
Warfarin	15.13	2.09–90.18	0.004	**–**	**–**	**–**
Heparin replacement	6.24	0.99–28.35	0.026	4.60	1.02–20.70	0.046
Lower third	7.14	1.55–66.93	0.004	6.67	1.43–31.00	0.016

With regard to the onset of post-ESD bleeding, 4 patients were in early-phase bleeding within 24 h of the procedure and 10 patients presented with late-phase bleeding after 24 h. The mean resection size was significantly higher in the early-phase bleeding group compared to the non-bleeding group (52.3 ± 26.1 mm vs 36.8 ± 26.1 mm; *p* < 0.05). The other factors did not differ between the early-phase bleeding group and the non-bleeding group. In the analysis of late-phase bleeding, the risk factors were the same as the overall post-ESD bleeding group.

### The influence of CSS administration for post-ESD bleeding

Regarding clinicopathological features, in the CSS group, the adenoma size, resection time, and rate of resection time over 100 min were significantly higher compared to the non-CSS group (*p* < 0.05) (Table 3). The differentiated type was significantly lower than non-CSS. Other factors were not significantly different between the groups. Overall, post-ESD bleeding was observed in 5.2% (9/174) of the CSS group and 3.8% (5/130) of the non-CSS group, with no significant difference between the two groups (*p* = 0.783) (Table 4). The median onset of post-ESD bleeding was day 2 (0–9) in the CSS group and day 0 (0–2) in the non-CSS group, which did not show a significant difference (*p* = 0.104). Among patients with Af, post-ESD bleeding was observed in 3 patients of the CSS group (21.4%), whereas in the non-CSS group, it was observed in 1 patient (10.0%). Among patients taking warfarin, post-ESD bleeding was observed in 3 patients of the CSS group (60.0%), whereas it was not observed in the non-CSS group (0%). Similarly, for patients with heparin replacement, post-ESD bleeding was observed in 3 patients of the CSS group (30.0%), while it was not observed in the non-CSS group (0%). Among patients with the tumor location in the lower third of the stomach, post-ESD bleeding was observed in 8 patients of the CSS group (10.7%), while it was observed in 4 patients (5.8%) in the non-CSS group. Thus, these data suggest that CSS administration was not effective for the prevention of post-ESD bleeding in the general population or in high-risk patients who were reviewed for this study.

**Table 3:** Clinicopathological features of the CSS and non-CSS groups

	CSS, *n* = 174	Non-CSS, *n* = 130	*p* value
Age, mean (SD)	72.6 (8.6)	74.1 (7.6)	0.110
Gender, *n* (%)			1
Male	103 (70.1)	94 (72.3)	
Female	44 (29.9)	36 (27.7)	
Co-morbidity (/lesion)			
Atrial fibrillation	14 (8.0)	10 (7.7)	1
Ischemic heart disease	5 (2.9)	7 (5.4)	0.373
Cerebrovascular disease	11 (6.3)	11 (8.5)	0.508
Chronic kidney disease on HD	3 (1.7)	1 (0.8)	0.638
Antithrombotic agent therapy, *n* (%)	(/lesion)		
Aspirin	12 (6.9)	12 (7.1)	0.521
Thienopyridine derivatives	12 (6.9)	8 (6.2)	1
Cilostazol	8 (4.6)	2 (1.5)	0.198
Warfarin	5 (2.9)	3 (2.3)	1
DOAC	18 (10.3)	8 (6.2)	0.220
others	11 (6.3)	13 (10.0)	0.284
Antithrombotic agent use, *n* (%)	53 (30.5)	39 (30.0)	1
Single	41 (23.6)	31 (23.8)	1
Doublet	9 (5.2)	6 (4.6)	1
Triplet	2 (1.1)	1 (0.8)	1
Heparin replacement	10 (5.7)	5 (3.8)	0.595
P-CAB	18 (10.3)	20 (15.4)	0.221
Tumor location, *n* (%)			
Lower third	75 (43.1)	69 (53.1)	0.104
Middle third	65 (37.4)	38 (29.2)	0.144
Upper third	34 (19.5)	22 (16.9)	0.654
Macroscopic type, *n* (%)			
Elevated	72 (41.4)	65 (50.0)	0.162
Flat/depressed	81 (46.6)	57 (43.8)	0.644
Mixed	21 (12.1)	8 (6.2)	0.113
Histology			
Adenoma	44 (25.3)	14 (10.8)	0.002
Differentiated type	123 (70.7)	110 (84.6)	0.006
Undifferentiated type	7 (4.0)	6 (4.6)	0.784
UL (+), *n* (%)	15 (8.6)	8 (6.2)	0.513
Depth			
SM2, *n* (%)	8 (4.6)	8 (6.2)	0.609
Resection size, mm(mean ± SD)	37.2 (12.9)	36.8 (12.4)	0.808
Resection size > 30 mm, *n* (%)	115 (66.1)	82 (63.1)	0.628
Tumor size, mm(mean ± SD)	17.4 (11.6)	15.6 (10.7)	0.177
Resection time, minutes(mean ± SD)	112.0 (74.5)	92.1 (65.2)	0.015
Resection time > 100 min, *n* (%)	81 (46.6)	44 (33.8)	0.034
*En bloc* resection, *n* (%)	167 (96.0)	128 (98.5)	0.309
R0 resection, *n* (%)	161 (92.5)	125 (96.2)	0.225
Intraoperative perforation, *n* (%)	5 (2.9)	0 (0)	0.074

**Table 4:** Post-ESD bleeding of the CSS and non-CSS groups

Post-ESD bleeding	CSS	Non-CSS	*p* value
Overall, *n* (%)	9/174 (5.2)	5/130 (3.8)	0.783
Median onset of post-ESD bleeding, day	2 (0–9)	0 (0–2)	0.104
Atrial fibrillation	3/14 (21.4)	1/10 (10.0)	0.615
Warfarin	3/5 (60.0)	0/3 (0)	0.196
Heparin replacement	3/10 (10.0)	0/5 (0)	0.505
Lower third	8/75 (10.7)	4/69 (5.8)	0.372

The chronological trend of post-ESD bleeding in the patients with CSS was analyzed before and after July 2017 because the guidelines for gastroenterological endoscopy in patients undergoing antithrombotic treatment was revised in Japan in July 2017 [11]. The rate of post-ESD bleeding was 6.7% (6/90) before July 2017 and 3.5% (3/84) after July 2017, which did not show a significant difference (*p* = 0.499). As a result, the preventive efficacy of CSS administration did not significantly change in terms of the chronological trend.

## Discussion

This is the first report to show the inefficacy of CSS administration for the prevention of the post-ESD bleeding, suggesting little merit of CSS administration for the prevention of the post-ESD bleeding.

In this study, the overall rate of post-ESD bleeding was 4.6%. The univariate analysis showed that Af, warfarin intake, heparin replacement, and tumor location in the lower third of the stomach were risk factors in the post-ESD bleeding group. The multivariate analysis showed that Af, heparin replacement, and tumor location in the lower third of the stomach were independent factors for post-ESD bleeding. It is well known that antithrombotic agents, including warfarin and heparin replacement, are risk factors for post-ESD bleeding [12]. Additionally, the lower third of the stomach is influenced by gastric peristalsis of antrum and bile or digestive enzyme reflux from the duodenum [13], so the location of the tumor in the lower third of the stomach was a high-risk factor for post-ESD bleeding. Then, we analyzed the rate of post-ESD bleeding among subgroups of patients who received CSS with such risk factors. The results showed that CSS administration was ineffective for preventing post-ESD bleeding, clearly indicating that CSS administration is not recommended for preventing post-ESD bleeding, even in the high-risk group.

In previous research, CSS administration has been shown to have a hemostatic effect in the total knee arthroplasty without impacting the fibrinolysis system [7, 14]. However, these orthopedic findings were based on excessive bleeding within 1 or 2 days post-operation. In gastric ESD, uncontrolled hemorrhage is rarely experienced due to advances in bleeding-preventive techniques and devices. The average value of decreased hemoglobin level was reported to be around 1.0 g/dL after ESD [15]. Therefore, the limited amount of blood loss during ESD might weaken the hemostatic effect of CSS. In addition, CSS is thought to approach the capillary vessels and provide a hemostatic effect [8]. Because exposed blood vessels on the artificial ulcer are usually ablated during ESD, CSS might not exert the hemostatic effect in such situations.

This study was associated with several limitations. First, this was a retrospective and a single-center study; however, the rate of post-ESD bleeding is similar to other reports [2]. Second, the sample size of patients with post-ESD bleeding was relatively small, even though the CSS group and non-CSS group included more than 130 patients each. Third, a background of the CSS group and the non-CSS group showed a significant difference in histology type, resection time, and rate of resection time over 100 min, although our analysis revealed that these factors were not related to the risk of the post-ESD bleeding (Tables 1 and 2).

In conclusion, CSS administration was not effective for the prevention of post-ESD bleeding in both the general population and in the higher-risk patients analyzed for this study. This suggests that the administration of CSS for post-ESD bleeding prevention may need to be reconsidered.

## Data Availability

The datasets supporting the conclusions of this article can be made available upon request.

## Abbreviations

CSS: Carbazochrome sodium sulfonate
ESD: Endoscopic submucosal dissection
OR: Odds ratio; 95% CI: 95% confidence interval
P-CAB: Potassium-competitive acid blocker
Af: Atrial fibrillation
PPI: Proton pump inhibitor
